# Huntington’s Disease: From Mutant Huntingtin Protein to Neurotrophic Factor Therapy

**Published:** 2011-06

**Authors:** Youssef Sari

**Affiliations:** *Department of Pharmacology, College of Pharmacy and Pharmaceutical Sciences, University of Toledo, Toledo, USA*

**Keywords:** neurotrophic factors, neurturin, mutant huntingtin protein, neuroprotection

## Abstract

Huntington’s disease (HD) is an inherited disorder characterized by neuronal dysfunction and degeneration in striatum and cerebral cortex. Although the signaling pathways involved in HD are not yet clearly elucidated, mutant huntingtin protein is a key factor in the induction of neurodegeneration. The mutant huntingtin protein alters intracellular Ca^2+^ homeostasis, disrupts intracellular trafficking and impairs gene transcription. In this review, I emphasize the effects of mutant huntingtin protein in Ca^2+^ handling and transcriptional factors. Transcriptional alterations are key factors in the deficits of several proteins involved in the cellular machinery. These proteins include neurotrophic factors such as brain-derived neurotrophic factor, fibroblast growth factor, glial-cell-line-derived neurotrophic factor, ciliary neurotrophic factor and neurturin that have been suggested to restore neuronal dysfunction, improve behavioral deficits and prolong the survival in animal models of HD. An understanding of the molecular pathways involved in neurodegeneration will shed light on the choice of neurotrophic factors targeting a specific neuronal population in HD and will consequently overcome behavioral deficits.

## INTRODUCTION

Huntington’s disease (HD) is an inherited disorder caused by expansion of a polyglutamine repeat within exon 1 of the huntingtin gene on chromosome 4 (Huntington’s Disease Collaborative Research Group) (1). The mutation of expanded CAG encodes the polyglutamine is the product for the mutant huntingtin protein, a key player in HD. Findings have shown that individuals carry from 6 to 35 CAG repeats are unaffected. The full penetrance occurs when the number of repeats exceeds 35; the gene encodes a version of huntingtin protein that leads to HD (2).

Alteration of the huntingtin protein is a key factor in the induction of dysfunction or neurodegeneration, both of which lead to HD. It is not clear how the mutated huntingtin protein induces neuronal dysfunction and neuronal degeneration. There is a possibility that HD is caused by accumulation of the polyglutamine fragments in the cytoplasm and nucleus. Consequently, the neuropathology includes neuronal atrophy in the cerebral cortex and the striatum, forebrain regions that process a wide range of information for behavioral output (3, 4). GABAergic medium spiny neurons are the major degenerated neurons in HD (3, 5, 6). It is noteworthy that the examination of postmortem brains from advanced HD patients shows degeneration of other brain regions, including the hippocampus, the angular gyrus, and the lateral tuberal nuclei of the hypothalamus (5, 7-9).

Currently, there is no treatment for HD; however, target compounds including neurotrophic factors have been considered to play a potentially significant role in neuroprotection. Studies have shown that there is a link between the mutant huntingtin protein and cellular proteins including neurotrophic factors (10-14). The regulation of the expression of neurotrophic factors and their receptors may play an important role in neuroprotection. Studies have used exogenous neurotrophic factors in HD animal models in order to establish trophic requirements of neurons. Among these neurotrophic factors are brain-derived neurotrophic factor (BDNF), fibroblast growth factor (FGF), glial cell line-derived neurotrophic factor (GDNF), ciliary neurotrophic factor (CNTF) and neurturin. These neurotrophic factors have been demonstrated to play a key role against the progression of HD and behavioral deficits found in animal models and patients. In this review paper, I discuss mutant huntingtin protein in HD with emphasis on its role in calcium homeostasis and apoptosis. The roles of mutant huntingtin protein in transcriptional factors and endocytosis-vesicluar transport are also discussed. Finally, I discuss the role of neurotrohpic factors in HD.

### Mutant huntingtin protein in HD

Mutant huntingtin protein can interact with other cellular proteins, leading to the progression of HD (15). The formation of neuronal intranuclear inclusions that contain mutant huntingtin protein causes neuronal degeneration in transgenic HD mouse models (16). The huntingtin protein itself is a cytoplasmic protein that interacts with vesicular and cytoskeletal proteins [for review, see ref. (10)]. Furthermore, studies demonstrated that huntingtin protein plays an important role in intracellular trafficking, including membrane recycling, clathrin-mediated endocytosis, neuronal transport and postsynaptic signaling (17-22). Thus, mutant huntingtin protein is likely to have an impact on a wide range of cellular functions. In addition, mutant huntingtin protein interacts with transcriptional regulatory proteins (23-25). Moreover, the expanded polyglutamine repeats facilitate the interactions of mutant huntingtin protein with huntingtin protein-associated proteins selectively expressed in the striatum and cortex. Among these proteins are calmodulin, huntingtin protein-associated protein (HAP-1), huntingtin protein-interacting proteins (HIP-1 and 2) and glyceraldehyde-3-phosphate dehydrogenase (GAPDH). The interactions of the mutant huntingtin protein with these proteins induce protein dysfunction and lead to toxicity characteristic of HD (16, 26-30). Thus, the mutant huntingtin protein may trigger a cascade of several intracellular pathways that cause death of some neurons, including medium spiny neurons, although huntingtin protein is expressed in all types of cells.

**Figure 1:**
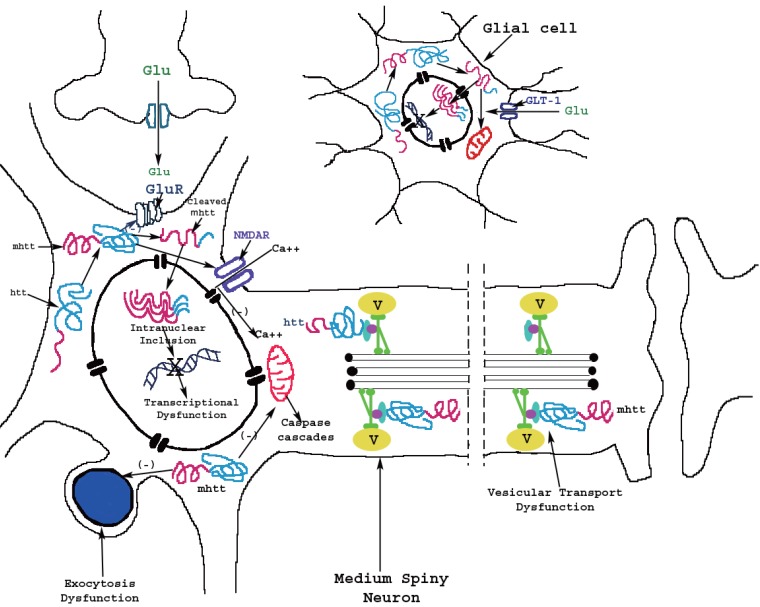
Model for mechanism of actions of mutant huntingtin protein (mhtt) in medium spiny neuron and glial cell in Huntington’s disease. The huntingtin protein is transformed to mhtt through unknown mechanism, genetically or environmentally, in both neuron and glial cell. There are several theories suggesting different actions of mhtt: 1) mhtt protein may interact with metabotropic (GluR) or ionotropic glutamatergic (NMDA) receptors and alters their function, 2) the mhtt protein may bind to cellular transport components and induce vesicular transport (V) or exocytosis dysfunction. Moreover, mhtt protein might be proteolytically cleaved in amino-terminal fragments which form β-sheet structures. In cytoplasm, the cleaved mhtt may interact with mitochondria and alters their function. In addition, the cleaved mhtt can enter the nucleus and forms intranuclear aggregates or intranuclear inclusions, which induce transcriptional dysfunction. Both mutant full-length and cleaved forms of htt may form soluble monomers, oligomers or large insoluble aggregates. Similar mechanism of the actions of cleaved mhtt is suggested to occur in glial cells. The cleaved mhtt may alter glutamatergic system such as glutamate transporter 1 (GLT-1) and consequently alters the uptake of glutamate.

There is little known about the role of glia in HD neuropathology. A previous study has shown that mutant huntingtin protein accumulates in nuclei of glial cells in the brain of HD mouse models (31). Interestingly, intranuclear mutant huntingtin protein in glial cells increases with age (Fig. 1) and was found to be correlated with disease progression in the R6/2 transgenic HD mouse model, which shows neuropathological symptoms around 6-8 weeks and often dies after 12 weeks of age (16).

**Effects of mutant huntingtin protein in calcium homeostasis.** Although the mechanism of action of mutant huntingtin protein is not clear, evidence has shown that it may induce mitochondrial dysfunction [for review, see ref. (32, 33)]. There is an interrelationship between mitochondrial dysfunction and dysregulation of transcriptional factors in HD [for review, see (34)]. Defects of mitochondrial respiratory chain activity have been shown in striatum of postmortem brains of patients suffering from HD and also in R6/2 HD mouse models (35, 36). Isolated mitochondria from lymphoblasts of HD patients and from brains of the YAC72 transgenic HD mouse (yeast artificial chromosome, length of polyglutamine is 72) have shown deficit in intracellular Ca^2+^ (37). Moreover, mutant huntingtin protein induces impairment of Ca^2+^ homeostasis in cloned striatal cells (38). Cells expressing mutant huntingtin protein show a reduction in mitochondrial Ca^2+^ uptake compared to wild type cells. The mutant huntingtin protein-induced lower Ca^2+^ loads were attenuated in the presence of ADP; the decreases in the uptake of Ca^2+^ were abolished in the presence of permeability transition pore inhibitors (38). Moreover, a fragment of mutant huntingtin protein may be directly bound with mitochondria (39) (Fig. 1); this has been shown at the ultrastructural level in the brain of YAC72 HD mouse model (37). Although the mechanism of action of mutant huntingtin protein in mitochondrial Ca^2+^ handling is still unknown, one possibility is that mutant huntingtin protein acts directly on the ion permeability of the mitochondrial membrane (37). Interestingly, several genes related to calcium signaling, including copine V, striatin, SCNβ4 and α-actinin2, are altered in the R6/1 transgenic HD mouse model (40). The highest level of gene expression is found in the subunit of the sodium channel, SCNβ4, with a decrease of its expression level in striatum of R6/1 HD mouse model compared to wild type. It has been suggested that sodium levels are directly dependent on intracellular Ca^2+^ levels through sodium-calcium exchanger (41). The reduction in SCNβ4 expression in R6/1 HD mice may have a dramatic effect on intracellular calcium accumulation (41). Decreases in Ca^2+^ signaling genes are found in HD mouse models (42). Interestingly, mutant huntingtin protein-induced alteration of calcium signaling was found to lead to apoptosis of medium spiny neurons in the YAC128 HD mouse model (43). Alteration of intracellular Ca^2+^ homeostasis may be a factor in the induction of apoptosis and consequent neurodegeneration in HD.

Several lines of evidence suggest that stimulation of glutamatergic receptors such as ionotropic [N-methyl-D-aspartate receptors, NMDA receptors (subunits NR1/NR2R)] and metabotropic (mGluR5) glutamate receptors alter Ca^2+^ homeostasis in striatal medium spiny neurons in HD models (43-45). The overstimulation of these receptors, through application of excess glutamate, results in mitochondrial Ca^2+^ overload leading to apoptosis of medium spiny neurons. Excess glutamate might be associated with impaired glutamate transport, as it was demonstrated in HD animal models from studies performed by us and others (46-49). Alteration in glutamate uptake might be linked to a deficit in one of the major glutamate transporters, a glial glutamate transporter 1 (GLT1), as it was demonstrated in our recent study (50). GLT1 is one of the proteins that might be altered by mutant huntingtin protein (Fig. 1).

Evidence indicates that perturbations of Ca^2+^ homeostasis may lead to excitotoxicity and, consequently, apoptosis (43, 51). Activation of NR1/NR2B NMDA receptors induces a Ca^2+^ influx; activation of mGluR5 leads to production of InsP3 and Ca^2+^ release via InsP3R1 (43, 52). The mutant huntingtin protein alters the Ca^2+^ handling in medium spiny neurons of HD mouse model through NMDA and mGlutamate receptors. This results in an overload of cytosolic Ca^2+^ along with an excess of mitochondrial Ca^2+^ storage, which lead to cytochrome c release into the cytosol, inducing apoptosis through activation of the caspase cascade (53-55). The caspases convey the apoptotic signal in a proteolytic cascade, with caspases cleaving and activating other caspases that subsequently degrade cellular targets, leading to cell death. Upon mitochondrial stress through disruption of Ca^2+^ homeostasis, the release of cytochrome c may interact with Apaf-1, causing self-cleavage and activation of caspase-9. The effector of caspase cascade, such as caspase-3, -6 and -7, is downstream of the activator caspases and acts to cleave various cellular targets. Recent studies demonstrated that caspase-6 is a key factor in the cleavage of mutant huntingtin protein in HD (56). Thus, cleavage of mutant huntingtin protein by caspase-6 is an important event in mediating neuronal dysfunction and possibly neurodegeneration. Interestingly, the cleavage of mutant huntingtin protein is dependent on the brain region. The cleavage at two N-terminal sites (A and B) was predominant in the cortex, whereas cleavage occurred at one N-terminal (A) and a C-terminal site in the striatum (57). In addition, inhibition of digestion of mutant huntingtin protein by both caspase-3 and 6 inhibitors was found to reduce apoptosis *in vitro*, which suggests that caspase inhibitors may be a key factor in the prevention of HD (58-60). Inhibitors of the apoptotic cascade may be used as a tool for prevention of cell death in HD (61). Moreover, the depletion of huntingtin protein has been found to induce activation of caspase-3, and the overexpression of this protein caused a reverse action of caspase-3, its inhibition (62). Huntingtin protein was found to interact with active caspase-3 at high affinity, but mutant huntingtin protein binds to caspase-3 at lower affinity. These findings suggest a mechanism whereby caspase-mediated huntingtin protein depletion results in an amplification cascade leading to further caspase-3 activation, resulting in neuronal dysfunction and neuronal death.

**Effects of mutant huntingtin protein in nucleus.** As shown in Fig. 1, mutant huntingtin protein impairs gene transcription through either intranuclear aggregate formation or sequestration to transcription factors that play a key role in HD [for review, see ref. (70, 71)]. Important transcriptional factors including p53, cAMP response-element binding protein (CREB)-binding protein (CBP), co-activator CA150, specificity protein 1 (SP1), co-activators TAFII130 and TFIID, and TATA-binding protein (TBP) can be recruited to intranuclear aggregates (72-75). There are interactions between these transcriptional factors and other associated proteins that may interact with SP1 in the regulation of gene expression. It has been demonstrated that huntingtin protein may strengthen the bridge between DNA-bound transcription factor SP1 and TFIID-associated proteins and consequently stimulate gene expression (72, 76, 77). The cAMP-responsive element (CRE) and CBP play a critical role in HD [for review see ref. (70)]. Alteration of CRE-regulated genes has been found in HD mouse models (78) and HD patients (79). The CBP and CRE-mediated transcription have been suggested to be affected by the coactivator TAFII130, which is also found in aggregates of CREB-dependent transcription (80). Additionally, mutant huntingtin protein may disrupt the interaction of SP1 and TAFII130 by formation of aggregates. Increased association of mutant huntingtin protein with SP1 has been found in brain extracts from HD patients. Consequently, the association of SP1 and TAFII130 was found to be reduced in brains of HD patients (72). Moreover, SP1 interacts with N-terminal huntingtin protein fragments in the nucleus of both transfected cells and in brains of HD mice (81). These findings suggest that shorter N-terminal huntingtin protein fragments, responsible for misfolding and aggregation, are more likely to bind SP1 and may inhibit its activity. Interestingly, this effect of huntingtin protein can be reversed by a molecular chaperone (Hsp40), which reduces the misfolding of mutant huntingtin protein.

There are other transcriptional factors that may interact with normal or mutant huntingtin protein in the nucleus. Among them, CA150 transcriptional factor has been found to interact with normal and mutant huntingtin protein (82). CA150 protein levels have been found to be increased in HD brain samples. There are also nuclear repressors that have been shown to interact with huntingtin protein, including N-CoR and C-terminal binding protein (CtBP) (73, 83). The mechanism of the repression appears to occur through the formation of a complex of repressor proteins including the N-CoR, mSin3, histone deacetylases and CtBP. The relocalization of repressor proteins in HD brains may alter transcription, which plays a role in HD neuropathology (83).

**Effects of mutant huntingtin protein in endocytosis and axonal vesicular transport.** Mutant huntingtin protein has been found to be involved in clathrin-mediated endocytosis. Dysregulation of endocytosis occurs with the interactions of mutant huntingtin protein with proteins that play a role in clathrin-mediated endocytosis (Fig. 1). Moreover, dysregulation of endocytosis is mediated through interactions of mutant huntingtin protein with its associated proteins, HIP1, HIP12, HIP14, PACSIN1 and SH3GL3, known as accessory factors in clathrin-dependent synaptic vesicle endocytosis (25, 30, 63-68). The interactions of mutant huntingtin protein with multiple accessory factors involve several steps that lead to dysregulation of clathrin-mediated endocytosis [for review, see ref. (12)]. Mutant huntingtin protein also is involved in vesicular transport processes in axons. In normal physiological situations, huntingtin protein and HAP1 are transported anterogradely and retrogradely along microtubules in axons (69). There is interaction of the complex huntingtin protein and HAP1 with dynactin, which influences the mobility of dynein in vesicular transport. Huntingtin protein and HAP1 stabilize dynein-dynactin complex of vesicles and consequently enable transport along microtubules in endocytosis processes (12). However, if huntingtin protein is mutated, dysfunctional interaction occurs, which leads to impairment of the anterograde and retrograde transport. Neurotrophic factors may be involved in this transport. Alterations of the transport of neurotrophic may be critical in cell survival.

### Role of neurotrophic factors in neuroprotection in HD

Neurotrophic factors play an important role in the prevention of apoptosis and cell differentiation. Neurotrophic factors are released by glial cells, neurons and other types of cells including endothelial and fibroblast cells. Deficiency of neurotrophic factors affects the neuroplasticity of the central nervous system (CNS) and consequently leads to neural death. A chronic deficit of neurotrophic factors involves several target tissues that may play a key role in degeneration of distinct neuronal populations in the adult (84). In addition, the deficiency of endogenous neurotrophic factors is considered critical for the progression of degeneration in neurodegenerative diseases, including HD (85-87).

**Neurotrophic or growth factors.** Neurotrophic factors are critical for cell differentiation, neuronal growth, and neuronal survival (88, 89). Among these neurotrophic factors, BDNF belongs to the neurotrophin family, which includes nerve growth factor (NGF), neurotrophin 3 (NT-3), and neurotrophin 4/5 (NT-4/5), neurotrophin-6 (NT-6), and neurotrophin-7 (NT-7) (90-92). The biological effects of neurotrophins (93-95) are mediated by high-affinity tyrosine kinase (Trk) receptors (96), although all neurotrophins also bind to a low-affinity receptor, p75^LNTR^ (97). NGF binds TrkA receptors, NT-4 and BDNF preferentially activate TrkB receptors, and NT-3 interacts with TrkC receptors. Neurotrophins are expressed by glial cells and neurons. Neuronal survival and/or neuronal differentiation also involve proteins other than neurotrophins, most notably the members of GDNF, which is a cloned member of the transforming growth factor (TGF) β-superfamily (98). Neurturin is another member of the TGFβ family that presents a GDNF-structurally-related neurotrophic action. Although GDNF and neurturin act through the same receptor complex (c-ret/GFRα), GDNF has binding preference for GFRα-1 and neurturin for GFRα-2 (99-101). CNTF is one of the neurotrophic factors that is distinct from neurotrophins in both structural and biological actions (102, 103). CNTF acts through CNTF receptor alpha and leukemia inhibitory factor receptor (104, 105). The roles of BDNF, FGF-2, GDNF, Neurturin, and CNTF are the factors that are involved in neuroprotection in HD.

**Brain Derived Neurotrophic Factor in HD.** BDNF is considered a particularly important trophic factor in HD. BDNF is produced by cortical neurons and transported to projection sites in the striatum, and it acts on striatal neuronal survival (106-109). In addition, the nigrostriatal pathway is considered another source of BDNF production and may play a key role in HD (110). The level of BDNF is decreased in the cortex and striatum of HD patients, which is possibly due to decrease in BDNF transcription (11, 13, 111). In animals, a reduction in the level of BDNF mRNA and its protein is found in cortex, striatum, hippocampus and/or cerebellum of transgenic HD mouse models (13, 14, 112-118).

Down-regulation of BDNF in HD mice is related to the length of CAG repeats and the levels of expression (119). This suggests that the decrease of the level of BDNF depends on both the number of CAG repeats and the level of expression of mutant huntingtin protein (119). However, expression of mutant huntingtin protein appears sufficient to alter the level of BDNF (13, 14). A recent study reported that HAP1 interacts with the prodomain of BDNF, however, this interaction is diminished with the presence of mutant huntingtin protein (120). Huntingtin protein is associated with vesicular structures and microtubules, which play an important role in intracellular trafficking (12). Mutant huntingtin protein has been found to impair the transport of BDNF (11, 121). These findings indicate that huntingtin protein promotes BDNF transport, and loss or mutation of huntingtin protein may contribute to deficit of BDNF, which leads to pathogenesis.

The cellular effects of BDNF are mediated through its receptor, TrkB. Reduction of TrkB receptors has been found in transgenic exon-1 and full-length knock-in HD mouse models as well as postmortem HD human brain (122, 123). Interestingly, the overexpression of mutant huntingtin protein may be required for the down-regulation of TrkB levels. Although, the precise mechanism through which mutant huntingtin protein decreases TrkB is still unclear, the expression of TrkB is regulated by CREB, which binds to the second cAMP-responsive element site of one of the TrkB gene promoters and then stimulates TrkB expression (124). Additionally, down-regulation of a regulatory BDNF gene, c-AMP-responsive element, was found in HD mice (13, 42, 113, 124-126). Moreover, mutant huntingtin protein may impair CREB-mediated transcription, which contributes to the reduction of TrkB expression found in HD. Thus, TrkB may contribute to the alteration of the neurotrophic effect in HD models. Together, these findings suggest that loss of trophic maintenance in animal HD models and in HD patients may be related to deficits in BDNF and to a decrease in TrkB expression.

Increasing the levels of BDNF in the cortico-striatal pathway might promote cell survival and, in turn, regulate genes that are transcriptionally disrupted in HD. Therapeutic approaches targeting an increase in BDNF might be a strategy to slow or prevent HD (10, 127). The effect of adenovirus-mediated transfer of the BDNF gene was studied in quinolinic-acid lesioned rat striatum. Intrastriatal injections of adenovirus encoding BDNF demonstrated neuroprotection of striatal neurons (128). Moreover, adenoassociated viral (AAV) vector-mediated gene delivery of BDNF induced a neuroprotective effect in quinolinic-acid treated rats. AAV-BDNF vector provides neuroprotection of striatal neurons in quinolinic-acid HD rats (129). These suggest that local BDNF gene delivery has therapeutic value for the treatment of neurodegeneration in HD. Moreover, another approach was developed to engineer cells that express BDNF and release it continuously (130-133). One of these studies demonstrated slight neuroprotection by the BDNF-secreting cells (130). However, the other studies using BDNF-secreting cells have shown significant improvement in motor performance and reduction in damaged striatal neurons (131-133). The authors of these studies suggested that the dose of BDNF plays a critical role in neuroprotection. The overexpression of BDNF may have a secondary side effect such as increase in neuronal excitability.

**Fibroblast Growth Factor in HD.** Among different types of FGF, FGF-2 type has been found to protect neurons exposed to toxins or excitatory amino acids (134). Other studies have shown that FGF-2 protects and exerts trophic effects on striatal neurons and stimulates proliferation of striatal neural stem cells (135-137). In addition, FGF-2 promotes neurogenesis, leads to neuroprotection and consequently prolongs survival of R6/2 transgenic mice (138). The increase of neurogenesis through the application of FGF-2 may be associated with the migration of nascent neurons in the subventricular zone toward the striatum, where these neurons become medium spiny neurons as the principal component to replace the lost in HD models. The use of FGF-2 may be considered a potential treatment strategy for the replacement of the lost of neurons in HD models.

**Glial cell line-Derived Neurotrophic Factor in HD.** GDNF is an important factor for the treatment of neurodegenerative diseases, including HD (139-142). GDNF prevents neurodegeneration of striatal calbindin- and parvalbumin-immunoreactive neurons in a lesion model of HD. The neuroprotection is specific to striatal, medium spiny neurons (141). GDNF is protective for striatal neurons of the indirect pathway, GABA/substance P neurons, which project to the internal segment of globus pallidus and/or substantia nigra pars reticulata (143).

Moreover, transplantation of mouse striatum infected with lentivirus expressing GDNF into the striatum of pre-symptomatic N171-82Q mice maintained motor function and prevented neuronal loss (144). Studies using AAV encoding GDNF have demonstrated a deficit of this trophic factor in N171-82Q transgenic HD mouse model (145). Viral delivery of GDNF induces structural and functional neuroprotection in this HD mouse model. In contrast, a study using R6/2 transgenic HD mouse model did not show any neuroprotective effects with GDNF viral delivery (146). This difference exists because R6/2 transgenic mice have human cDNA that encodes mutant huntingtin protein with a larger number of repeats than those of N171-82Q transgenic mice. Importantly, GDNF treatment fails its neuroprotective effect when it is applied after the time when the inclusions of mutant huntingtin protein have been formed in the striatum (146). Thus, GDNF might be efficient when delivered at the time of the expression of mutant huntingtin protein. The criteria of the application of this trophic factor in a specific age of the progression of HD may apply to all neurotrophic factors.

**Neurturin in HD.** Neurturin is a neurotrophic factor of the TGFβ family structurally related to GDNF. High doses of neurturin have been found to protect striatal neurons from death in both kainic-acid and quinolinic-acid HD models (147). The neuroprotective effect of neurturin requires repeated injections to maintain high levels within the striatum for a long period of time (148). A recent study has used AAV vector encoding for the trophic factor neurturin in 3-nitropropionic acid (3NP)-treated rats. AAV-neurturin-3NP application induces neuroprotection as compared to saline-3NP-treated rats (149). The advantage of AAV vectors is that they can have long-term gene expression. Thus, the neuroprotective effect of neurturin may persist for several years without repeated manipulations or surgeries (149-151). Another approach was used for neuroprotection of striatal neurons in quinolinic-acid treated rats (HD model). A fibroblast cell line engineered to over-express neurturin was injected into adult rat striatum one day before quinolinic acid injection; grafting of the neurturin-secreting cell line showed a more specific and efficient trophic effect on striatal neurons of the indirect pathway (152).

The neurotrophic action of neurturin is more specific to certain striatal neuronal populations. In quinolinic-acid HD model, neurturin selectively protects striatal neuron projections of the indirect circuit, which is the first to enter apoptosis stage in HD patients (153, 154). Neurturin selectively protects the striatopallidal neurons expressing glutamic acid decarboxylase and preproenkephalin (155). This suggests that neurturin protects only GABAergic and enkephalinergic neurons that project to the external segment of the globus pallidus [for review, see (143)]. Therefore, these results suggest that neurturin is considered a candidate for the treatment of HD.

**Ciliary neurotrophic factor in HD.** CNTF is a member of the alpha-helical IL-6 cytokine superfamily with neurotrophic actions in the peripheral and CNS. CNTF stimulates gene expression, cell survival and differentiation in several types of neurons including GABAergic and cholinergic neurons and also plays a role in oligodendrocyte maturation (156, 157). CNTF provides neuroprotection for striatal neurons; it is suggested to be a potential therapeutic agent for HD (158, 159).

Application of CNTF in CNS has been a challenge due to its difficult accessibility into the brain via systemic injection. CNTF is a large protein that does not cross the blood brain barrier. Thus, local delivery using polymer-encapsulated cells engineered to secrete CNTF shows pre-clinical and clinical success for the delivery of this trophic factor to the CNS (159-163). Moreover, the protective effect of encapsulated cells producing human CNTF was successful in degenerating striatal neurons as well as neurons in the cerebral cortex. In HD monkeys, CNTF prevents degeneration of striatal and cortical neurons (163). A neuroprotective effect was observed in HD patients using polymer-encapsulated cells engineered to release human CNTF in the striatum. The clinical phase I studies show a promising neuroprotective effect of CNTF for the treatment of HD patients (160).

## CONCLUSION

Although the mechanisms of neurodegeneration in HD remain unclear, mutant huntingtin protein is suggested to alter intracellular trafficking including membrane recycling, clathrin-mediated endocytosis and neuronal transport. Deficit of neurotrophic factor levels in HD mouse models may be linked to direct action of mutant huntingtin protein in the transport of these trophic factors. In addition, mutant huntingtin protein has been demonstrated to interact with cellular transcriptional regulatory proteins, some of which are neurotrophic factors.

Understanding the molecular mechanism of actions of mutant huntingtin protein may underlie expectations for the discovery of drugs that can lead to neurorestoration or neuroprotection in which growth of new axons, dendrites, and synapses might be consequences of functional improvement. These mechanisms might be of great interest for patients who already have the diseases or, in some cases, where the disease is in progression. Neurotrophic factors are considered one of the therapeutic tools for the treatment of HD to overcome neurodegeneration and behavioral abnormalities.

## ABBREVIATIONS

HD: Huntington’s disease
BDNF: Brain-derived neurotrophic factor
FGF: Fibroblast growth factor
GDNF: Glial cell line-derived neurotrophic factor
CNTF: Ciliary neurotrophic factor
HAP-1: Huntingtin protein-associated protein
HIP-1: Huntingtin protein-interacting protein 1
GAPDH: Glyceraldehyde-3-phosphate dehydrogenase
YAC: Yeast artificial chromosome
NMDA: N-methyl-D-aspartate receptor
mGluR5: metabotropic glutamate receptor 5
Apaf-1: Apoptotic protease activating factor 1
CREB: cAMP response-element binding protein
CBP: cAMP binding protein
SP1: Specificity protein 1
CtBP: C-terminal binding protein
CNS: Central nervous system
NGF: Nerve growth factor
NT-3: Neurotrophin 3
Trk: Tyrosine kinase
TGF-β: Transforming growth factor β
AAV: Adenoassociated viral
3NP: 3-nitropropionic acid
